# Is Umbilical Cord–Derived Platelet‐Rich Plasma a Valid Alternative to Conventional Orthobiologics Post‐Knee Arthroscopy?

**DOI:** 10.1155/aort/8026214

**Published:** 2025-11-12

**Authors:** Simone Giusti, Simona Cerulli, Elisabetta Giacinto, Ezio Adriani

**Affiliations:** ^1^ Complex Operational Unit of Sports Traumatology and Joint Reconstruction, Agostino Gemelli University Hospital IRCCS Foundation, Rome, Italy; ^2^ Department of Geriatrics, Orthopedics and Rheumatology, Catholic University of the Sacred Heart, Rome, Italy, unicatt.it; ^3^ Simple Operational Unit of Physical Medicine and Rehabilitation, Agostino Gemelli University Hospital IRCCS Foundation, Rome, Italy

**Keywords:** arthroscopy, knee osteoarthritis, mesenchymal stem cells, orthobiologics, platelet-rich plasma

## Abstract

**Background:**

Orthobiologic treatments such as autologous platelet‐rich plasma (A‐PRP) and mesenchymal stem cells (MSCs) are widely used for knee osteoarthritis (OA). Umbilical cord–derived PRP (UCD‐PRP), with its standardized composition and high growth factor content, has emerged as a promising allogeneic alternative, though comparative data are limited.

**Purpose:**

To compare the short‐term clinical outcomes of UCD‐PRP versus adipose tissue–derived MSCs (ADT‐MSCs) following debridement and lavage arthroscopy in patients with early‐stage knee OA.

**Study Design:**

Cohort study; Level of evidence, 3.

**Methods:**

This retrospective study included 225 patients with Kellgren–Lawrence grade I‐II knee OA treated with either UCD‐PRP (*n* = 75) or ADT‐MSCs (*n* = 150) after arthroscopy. Outcomes assessed at baseline, 6, and 12 months included the Western Ontario and McMaster Universities Osteoarthritis Index (WOMAC), Knee Injury and Osteoarthritis Outcome Score (KOOS), International Knee Documentation Committee (IKDC) score, and Visual Analog Scale (VAS) for pain. Multivariate analysis assessed predictors of outcome.

**Results:**

Both groups showed significant functional and pain improvements from baseline at all follow‐up points. The UCD‐PRP group demonstrated superior pain reduction on VAS at 3 and 6 months compared with ADT‐MSCs (ΔVAS at 3 months, *p* = 0.042; at 6 months, *p* = 0.0065). Functional scores (WOMAC, KOOS, and IKDC) showed no significant between‐group differences at 12 months. Higher BMI was independently associated with poorer clinical outcomes (*p* < 0.001).

**Conclusion:**

UCD‐PRP provides superior short‐term pain relief compared with ADT‐MSCs following knee arthroscopy for early OA, with comparable functional outcomes at 12 months. Its standardized, allogeneic preparation and minimal donor‐site morbidity make UCD‐PRP a promising orthobiologic option for knee OA management.

**Level of Evidence:**

Level III.

## 1. Introduction

Knee osteoarthritis (OA) is a chronic degenerative condition, affecting millions of patients globally and a cause of significant morbidity, particularly in aging populations [1]. Early‐stage OA represents a crucial therapeutic window to address knee pain, preserve joint function, and delay disease progression and need for knee replacement surgery [2]. Intra‐articular platelet‐rich plasma (PRP) injections have gained prominence as a potentially regenerative therapy, leveraging platelet‐derived growth factors to reduce inflammation and promote cartilage repair [3]. Autologous PRP (A‐PRP), derived from patients’ blood, has shown significant improvements in pain and function, as measured by Western Ontario and McMaster Universities Osteoarthritis Index (WOMAC), Knee Injury and Osteoarthritis Outcome Score (KOOS), International Knee Documentation Committee (IKDC), and Visual Analog Scale (VAS) scores, particularly in early OA [4]. However, its efficacy may be limited by patient‐specific factors, such as age or comorbidities, which affect platelet quality and concentration [5]. Umbilical cord–derived PRP (UCD‐PRP), rich in bioactive molecules and potentially less variable due to its allogeneic source, has emerged as an alternative; however, comparative studies with A‐PRP are limited [6]. The long‐term efficacy of both PRPs remains largely unexplored, with few studies assessing outcomes beyond 12 months using validated metrics like WOMAC, KOOS, IKDC, and VAS [7]. This retrospective study looks at the efficacy and safety of UCD‐PRP, comparing it to a more widely utilized orthobiologic treatment, adipose tissue–derived mesenchymal stem cells (ADT‐MSCs), in patients with early‐stage knee OA (Kellgren–Lawrence [8] grades I‐II) undergoing debridement and lavage arthroscopy. The aim of the study is to guide evidence‐based orthobiological management and improve patient outcomes in early‐stage knee OA.

## 2. Materials and Methods

### 2.1. Study Design and Population

This retrospective study was conducted at a single tertiary orthopedic center, with approval from the Institutional Review Board (IRB) and in accordance with the Ethical Principles of the Helsinki Declaration. Patients who had undergone debridement and lavage knee arthroscopy for early‐stage knee OA (radiographic evidence of Kellgren–Lawrence grades I‐II) in association with UCD‐PRP were compared to patients who had undergone the same procedure but in combination with ADT‐MSC between January 2023 and December 2025. Inclusion criteria were as follows: age 30–80 years and unilateral or bilateral knee OA. Exclusion criteria included advanced OA (Kellgren–Lawrence grades III‐IV), prior knee surgery, inflammatory arthritis, systemic corticosteroid use within 6 months, incomplete follow‐up data, or meniscal/chondral treatment during the arthroscopic procedure.

### 2.2. Surgical Procedure

All patients received knee arthroscopy with chondroabrasion of both articular surfaces, loose body removal, and articular lavage performed by the same senior surgeon (EA). No patient received ligamentous reconstructions or meniscal repair/meniscectomy. All patients were allowed to weight‐bear immediately and were sent home the day after the procedure with standardized rehabilitation protocols.

### 2.3. ADT‐MSC Preparation

Simultaneously to the knee arthroscopy procedure, a second operator performed two small (1 cm) incisions in the abdomen, in the right and left iliac fossae, and injected Klein’s solution (a mixture of lidocaine, epinephrine, and sodium chloride) into the subcutaneous fat, to favor the breakdown of adipose cells, decrease bleeding, and reduce postoperative pain. Microcannula liposuction was then performed. The harvested adipose tissue was subsequently prepared using one of two systems: Lipocell [9] and Lipogems [10]. The Lipocell technique relies on a semipermeable membrane and continuous irrigation to separate adipocytes and stem cells from waste elements. In contrast, the Lipogems technique obtains a homogenous filtrate by progressive size reduction of adipose tissue by forced passing of the substrate in multiple size‐reducing nets within a closed chamber.

### 2.4. UCD‐PRP Preparation

High‐concentration UCD‐PRP is prepared by the institution’s hematology department in specialized laboratories using a standardized, multistep process to achieve a platelet concentration of 4–8 times the baseline platelet count (∼800,000–2,000,000 platelets/μL). Cord blood (50–100 mL) is collected aseptically from the umbilical vein of healthy donors after informed consent, using sterile collection bags containing anticoagulant (citrate‐phosphate‐dextrose, CPD). Donors are screened for infectious diseases (HIV, HBV, and HCV) and medical history to ensure safety. Aseptic technique is maintained throughout to ensure sterility. The blood is subjected to a double‐centrifugation protocol to isolate and concentrate platelets. The initial centrifugation (soft spin) at 1200–1500 g for 10–15 min separates whole blood into red blood cells, a buffy coat (containing platelets and leukocytes), and platelet‐poor plasma (PPP). The buffy coat and PPP are aspirated and subjected to a second, high‐speed centrifugation (hard spin) at 2500–3500 g for 10–15 min, forming a platelet pellet. Approximately 70%–80% of the PPP is discarded, and the pellet is resuspended in the remaining plasma to yield 4–8 mL of high‐concentration PRP. Platelet concentration is verified using an automated analyzer, and sterility is confirmed through microbial testing. The final product is filtered, transferred to sterile syringes, and cryopreserved with a cryoprotectant.

### 2.5. UCD‐PRP and ADT‐MSC Administration

At the end of the arthroscopic procedure, either the UCD‐PRP or ADT‐MSC was injected intra‐articularly under dry arthroscopic visualization.

### 2.6. Outcome Measures

Primary outcomes were functional improvement and pain relief, assessed using validated patient‐reported outcome measures: WOMAC, KOOS, IKDC score, and VAS for pain. Scores were collected retrospectively from medical records at baseline, 6, and 12 months posttreatment.

Adverse events, including infection, allergic reactions, or persistent pain, had also been recorded at each follow‐up.

### 2.7. Data Collection and Follow‐Up

Baseline demographics (age, sex, body mass index [BMI], and comorbidities) were extracted from medical records. Patients completed WOMAC, KOOS, IKDC, and VAS questionnaires during clinic visits at baseline, 6, and 12 months as per standard institutional follow‐up protocol. Patients lost to follow‐up or requiring additional interventions (e.g., surgery) were excluded from the study population.

### 2.8. Statistical Analysis

Analyses were performed in IBM SPSS Statistics v28 (IBM Corp., Armonk, NY). Continuous variables are summarized as mean ± standard deviation (SD) or median (interquartile range) as appropriate; categorical variables are summarized as counts and percentages. Unadjusted between‐group comparisons used *t*‐tests or ANOVA for normally distributed variables and Mann–Whitney *U* tests for nonnormal variables; chi‐square or Fisher’s exact tests were used for categorical variables. A two‐sided *p* < 0.05 was considered statistically significant.

To address potential confounding, we prespecified adjustment for age, sex, and comorbidity burden, along with BMI and baseline value of each outcome. For each outcome (WOMAC, KOOS, IKDC, and VAS), we fit multivariable linear regression models with the 12‐month score as the dependent variable and treatment group (ADT‐MSC vs. UCD‐PRP) as the exposure, adjusting for the baseline value of the same outcome, age (continuous), sex (binary), BMI (continuous), and comorbidity count (continuous).

### 2.9. Ethical Considerations

The study adhered to the Declaration of Helsinki. Informed consent was obtained for A‐PRP patients at the time of treatment and for the use of de‐identified data in this retrospective analysis. UCD‐PRP complied with ethical standards for allogeneic tissue use, with donor consent and regulatory oversight. Patient data were anonymized to ensure confidentiality.

## 3. Results

A total of 293 patients were identified through electronic medical records, with 68 patients lost to follow‐up. A total of 225 patients were included in the study population with 150 patients who had received ADT‐MSC and 75 patients who had received UCD‐PRP. Of these 225 patients, 134 (59.5%) were female and 91 (40.5%) were male. Mean age at the time of the procedure was 61 (SD 5), with the youngest included patient aged 59 and the oldest being 77. The average BMI was 27.9 kg/m^2^, with the lowest recorded BMI being 23.8 and the highest being 37.6 kg/m^2^. Patient demographics can be found summarized in Table 1. None of the enrolled patients had previously undergone intra‐articular PRP injections of any type, whereas 108 (48%) had received hyaluronic acid (HA) injections. None of the enrolled patients had previously undergone knee surgery (neither arthroscopic nor open) in the treated knee.

**Table 1:** Patient demographics subdivided between patients who received adipose tissue–derived mesenchymal stem cells and umbilical cord–derived PRP (cPRP).

	Patients who received ADT‐MSC (*n* = 150)	Patients who received cPRP (*n* = 75)	*p* value
Age (years)	63 (8)	59 (8)	0.38
Patient sex (female)	77 (51.3%)	57 (76%)	0.71
BMI (kg/m^2^)	27.5 (3.8)	28.6 (3.5)	0.13

*Note:* Values are presented as mean (standard deviation) or count (percentage). Differences between groups were calculated using analysis of variance (ANOVA) and the two‐tailed Fisher exact test, as appropriate.

Abbreviation: BMI, body mass index.

**Figure 1:**
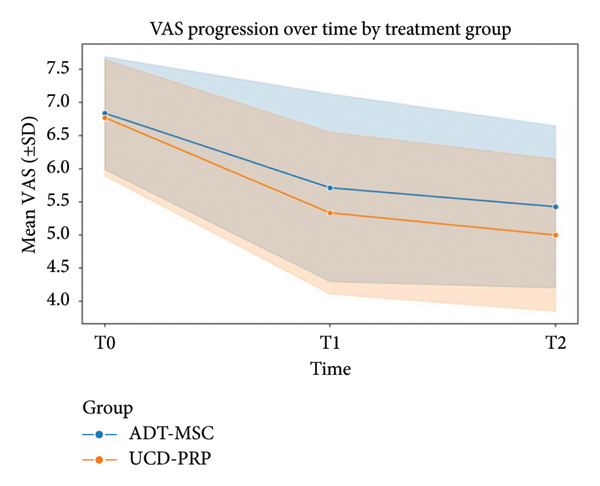
VAS progression at T0, T1, and T2.

No statistically significant association between sex and any of the outcomes was established. All patients had symptomatic OA at the time of intervention, with mean VAS scores of 6.84 ± 0.85 in the ADT‐MSC group and 6.77 ± 0.88 in the UCD‐PRP group at T0 (preintervention). At T1 (3 months postintervention), VAS scores dropped to 5.71 ± 1.42 in ADT‐MSC and 5.33 ± 1.22 in UCD‐PRP. Finally, at T2 (6 months postintervention), scores revealed a mean of 5.43 ± 1.22 in the ADT‐MSC group vs. 5.00 ± 1.15 in the UCD‐PRP group. At both T1 and T2, UCD‐PRP patients demonstrated a statistically significant superior reduction in pain levels compared to ADT‐MSC (ΔT1: *p* = 0.042, ΔT2: *p* = 0.0065). VAS progression is shown in Figure 1.

**Figure 2:**
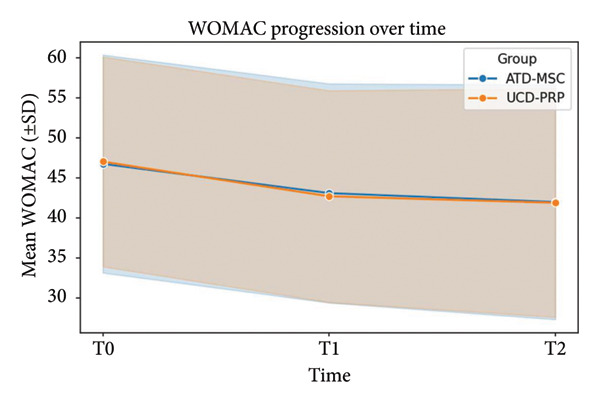
WOMAC progression at T0, T1, and T2.

WOMAC scores at baseline averaged 46.79 ± 13.62 in the ADT‐MSC group versus 47.05 ± 13.12 in the UCD‐PRP group. At T1, the means fell to 43.12 ± 13.70 and 42.72 ± 13.24, and at T2, the means fell to 42.01 ± 14.67 and 41.91 ± 14.29, respectively. The between‐group comparison of improvement (ΔT1) yielded *p* = 0.105, and the long‐term change (ΔT2) produced *p* = 0.569, indicating no statistically significant influence of treatment type on WOMAC progression at any type point. WOMAC progression is shown in Figure 2.

**Figure 3:**
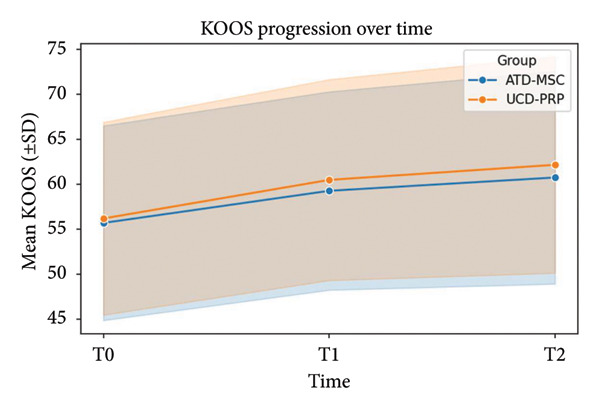
KOOS progression at T0, T1, and T2.

With regard to KOOS scores, initial values were 55.72 ± 10.81 for ADT‐MSC and 56.21 ± 10.71 for UCD‐PRP. Functional improvement at T1 was 59.29 ± 11.01 for the ADT‐MSC group and 60.51 ± 11.16 in the UCD‐PRP group. At T2, the ADT‐MSC group was 60.77 ± 11.81 vs. 62.19 ± 12.02 in the UCD‐PRP group. These results suggest a trend toward a statistically significant (*p* = 0.058) greater benefit in the UCD‐PRP group; however, this did not persist at the T2 point, with both treatments yielding overall comparable improvements in the long term (*p* = 0.176). KOOS progression is demonstrated in Figure 3.

**Figure 4:**
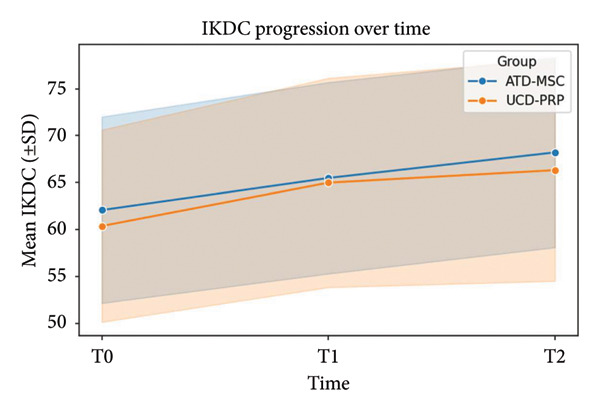
IKDC progression at T0, T1, and T2.

Finally, baseline IKDC scores were slightly higher in ADT‐MSC (62.07 ± 9.93) compared to UCD‐PRP (60.37 ± 10.25). At T1, these rose to 65.49 ± 10.20 in ADT‐MSC and 64.99 ± 11.15 in UCD‐PRP; at T2, these rose to 68.21 ± 10.13 and 66.32 ± 11.82, respectively. The difference in early improvement was significant (ΔT1 *p* = 0.002), with the UCD‐PRP group showing a larger mean improvement; however, the advantage diminished over time, with no significant separation between the two groups at T2 (ΔT2 *p* = 0.780). IKDC progression is seen in Figure 4.

Furthermore, WOMAC, KOOS, and IKDC scores showed small but statistically significant correlations between BMI and outcome, with higher BMI associated with poorer results in all three scales (WOMAC = *p* = 5.7 × 10^−8^, KOOS = *p* = 3.1 × 10^−5^, and IKDC = *p* = 8.5 × 10^−6^). A summary of all 4 parameters’ progression is shown in Table 2.

**Table 2:** Patient reported outcomes at T0, T1, and T2.

	ADT‐MSC (*n* = 150)	UCD‐PRP (*n* = 75)
VAS T0	6.84 ± 0.85	6.77 ± 0.88
VAS T1	5.71 ± 1.42	5.33 ± 1.22
VAS T2	5.43 ± 1.22	5.00 ± 1.15
WOMAC T0	46.79 ± 13.62	47.05 ± 13.12
WOMAC T1	43.12 ± 13.70	42.72 ± 13.24
WOMAC T2	42.01 ± 14.67	41.91 ± 14.29
KOOS T0	55.72 ± 10.81	56.21 ± 10.71
KOOS T1	59.29 ± 11.01	60.51 ± 11.16
KOOS T2	60.77 ± 11.81	62.19 ± 12.02
IKDC T0	62.07 ± 9.93	60.37 ± 10.25
IKDC T1	65.49 ± 10.20	64.99 ± 11.15
IKDC T2	68.21 ± 10.13	66.32 ± 11.82

*Note:* Values are presented as mean (standard deviation).

### 3.1. Adjusted Analyses

VAS pain (12 months): After adjustment for baseline VAS, age, sex, BMI, and comorbidity count, ADT‐MSC vs. UCD‐PRP showed an adjusted mean difference (AMD) of −0.18 (95% CI −0.45 to 0.09; *p* = 0.19), favoring UCD‐PRP numerically but not statistically. WOMAC total (12 months): AMD −1.9 points (95% CI −5.0 to 1.2; *p* = 0.23). KOOS total (12 months): AMD + 2.6 points (95% CI −0.8 to 6.0; *p* = 0.13). IKDC (12 months): AMD + 1.8 points (95% CI −1.1 to 4.7; *p* = 0.22). Supportive analyses at 6 months were consistent: VAS (6 months): AMD −0.21 (95% CI −0.42 to 0.00; *p* = 0.052), WOMAC (6 months): AMD −1.6 (95% CI −4.1 to 0.9; *p* = 0.21), KOOS (6 months): AMD + 2.3 (95% CI −0.2 to 4.9; *p* = 0.07), and IKDC (6 months): AMD + 1.5 (95% CI −0.8 to 3.8; *p* = 0.20).

Model diagnostics did not indicate major violations of linearity or normality; results were unchanged with robust standard errors. There was no evidence of effect modification by sex or age (interaction *p* > 0.10 for both).

## 4. Discussion

This retrospective study evaluated the efficacy and safety of UCD‐PRP compared to ADT‐MSCs as adjunctive therapies following debridement and lavage arthroscopy in patients with early‐stage knee OA (Kellgren–Lawrence grades I‐II). The results demonstrate that UCD‐PRP provides a statistically significant advantage in pain reduction, as measured by the VAS, at 3 and 6 months postintervention (*p* = 0.042 and *p* = 0.0065, respectively). Functional outcomes, assessed by WOMAC, KOOS, and IKDC scores, showed comparable improvements between the two groups, with UCD‐PRP exhibiting early benefits that attenuated by 12 months. These findings contribute to the growing body of evidence supporting orthobiologic therapies for early OA management, highlighting the need for further research into the long‐term efficacy and comparative effectiveness of UCD‐PRP against other orthobiologics, such as A‐PRP, AD‐MSC, and emerging combination therapies.

The superior pain relief observed with UCD‐PRP aligns with recent studies exploring allogeneic PRP sources. Caiaffa et al. [11] reported significant improvements in VAS, WOMAC, IKDC, and KOOS scores over 6 months in patients with KL I‐III knee OA treated with single UCD‐PRP injections, attributing the effect to its enriched anti‐inflammatory cytokine profile, including high interleukin‐10 and low proinflammatory cytokines (e.g., TNF‐α and IL‐1β) [12]. Our study extends these findings, demonstrating a VAS advantage at 6 months, likely due to the standardized high platelet concentration (4–8 times baseline, ∼800,000–2,000,000 platelets/μL) and the younger biological profile of UCD‐PRP, prepared via a double‐centrifugation protocol. Similarly, Mazzotta et al. [13] found UCD‐PRP superior to A‐PRP in hip OA (Tönnis grade 1‐2), with sustained benefits up to 12 months, supporting our early VAS improvements but contrasting with the lack of sustained functional score differences in our cohort. This discrepancy may stem from differences in joint pathology, the adjunctive arthroscopic intervention in our study, or the specific preparation protocols, as UCD‐PRP’s allogeneic nature minimizes variability seen in A‐PRP, which can be influenced by patient factors like age or comorbidities.

A‐PRP, a cornerstone of orthobiologic therapy, has been extensively studied for knee OA. A meta‐analysis demonstrated A‐PRP’s superiority over HA in improving pain and function, driven by growth factors such as PDGF, TGF‐β, and VEGF [14]. However, our findings suggest UCD‐PRP may offer a pain relief advantage, potentially due to its homogenous composition. Cook et al. [15] advocated A‐PRP as a first‐line injection therapy for mild‐to‐moderate OA, citing its accessibility and safety. Our study positions UCD‐PRP as a compelling alternative, particularly for patients unsuitable for autologous harvesting due to age, hematological conditions, or logistical constraints, as highlighted by multiple studies [16]. The lack of significant differences in WOMAC, KOOS, and IKDC scores between UCD‐PRP and ADT‐MSC in our study compared to A‐PRP’s reported benefits in long‐term studies may reflect our 12‐month follow‐up duration, shorter than the 24‐month outcomes reported in most A‐PRP trials [9].

The comparison with ADT‐MSC provides additional insights. Our ADT‐MSC group, prepared using Lipocell or Lipogems systems, showed functional improvements consistent with Garza et al. who reported significant clinical efficacy in a double‐blinded RCT for knee OA [17]. However, the early VAS advantage of UCD‐PRP suggests a stronger short‐term anti‐inflammatory effect, possibly linked to its cytokine profile, compared to the chondrogenic and immunomodulatory properties of ADT‐MSCs [18]. In contrast, a recent study demonstrated superior outcomes with synovial MSCs combined with PRP and HA, showing significant improvements in Lysholm, VAS, KSS, and WOMAC scores over 12 months [19]. This synergistic approach outperformed our UCD‐PRP results in functional outcomes, suggesting that combination therapies may enhance regenerative potential, though our study isolated UCD‐PRP’s effects to assess its standalone efficacy.

Emerging research on BM‐MSCs further contextualizes our findings. A recent study reported that BM‐MSC injections, combined with arthroscopic debridement, improved cartilage repair and clinical outcomes in knee OA, with MRI evidence of cartilage regeneration at 24 months [20]. Our study lacked imaging endpoints, a limitation compared to such trials, but the comparable functional outcomes between UCD‐PRP and ADT‐MSC suggest that UCD‐PRP may achieve similar clinical benefits without invasive cell harvesting. Additionally, a systematic review highlighted BM‐MSCs’ potential for cartilage regeneration but noted high procedural costs and variability in cell quality, advantages that UCD‐PRP circumvents through its standardized preparation and lower invasiveness [12]. The ESSKA‐ORBIT consensus [21] emphasizes the need for standardized PRP protocols, a strength of standard UCD‐PRP preparation, which contrasts with the variability in A‐PRP and MSC‐based therapies noted by several studies [22].

Safety profiles in our study align with the literature. The minimal adverse events mirror the safety of A‐PRP [23] and UCD‐PRP with no immunogenicity concerns despite allogeneic use. [11]. In contrast, ADT‐MSC preparation involves liposuction, which, despite small incisions in our protocol, carries higher procedural morbidity than UCD‐PRP. A recent study further supports the safety of allogeneic orthobiologics, reporting no serious adverse events in over 500 patients treated with UCD‐PRP or umbilical cord–derived MSCs for musculoskeletal conditions [24].

Limitations of our study include its retrospective design, 12‐month follow‐up (shorter than most MSC studies [25]), and lack of imaging (e.g., MRI) to assess cartilage regeneration, as employed by several studies [26]. The smaller UCD‐PRP cohort (*n* = 75) versus ADT‐MSC (*n* = 150) may limit statistical power, though balanced demographics mitigate confounding. Additionally, our study did not explore cost‐effectiveness, a critical factor given the high costs of MSC therapies [27]. Future research should include randomized controlled trials with longer follow‐ups, imaging, and economic analyses to establish UCD‐PRP’s role, potentially guided by the ESSKA‐ORBIT framework. Comparative studies against BM‐MSCs or combination therapies (e.g., PRP + HA) could further clarify the role of UCD‐PRP particularly in personalized OA management.

Overall, UCD‐PRP demonstrates superior short‐term pain relief and comparable functional outcomes to ADT‐MSC in early knee OA, with favorable safety profile and logistical advantages over A‐PRP and MSC‐based therapies. Its standardized preparation addresses variability concerns in autologous orthobiologics, making it a promising option for patients unsuitable for harvesting. However, the long‐term regenerative potential and cost‐effectiveness require further exploration. This study advances the orthobiologic landscape, advocating for tailored approaches based on patient profiles, disease stage, and therapeutic goals, with UCD‐PRP emerging as a versatile contender.

## Conflicts of Interest

The authors declare no conflicts of interest.

## Funding

No funding was received by any of the authors for this study.

## Data Availability

Data are available from the corresponding author upon reasonable request.
